# Inequalities and Differences in Health Status of Pre- and Perinatal Periods in Hungarian Long-Term Series Analysis (1997–2019) †

**DOI:** 10.3390/children11111373

**Published:** 2024-11-12

**Authors:** Ágota M. Kornyicki, Anita R. Fedor

**Affiliations:** 1 Department of Methodology and Prevention for Health Visitors, Institute of Health Sciences, Faculty of Health Sciences, University of Debrecen, H-4400 Nyíregyháza, Hungary; 2 Department of Health Promotion, Szabolcs-Szatmár-Bereg County Teaching Hospital, H-4400 Nyíregyháza, Hungary; 3 Department of Social Sciences and Social Work, Institute of Social Sciences, Faculty of Health Sciences, University of Debrecen, H-4400 Nyíregyháza, Hungary; fedor.anita@etk.unideb.hu

**Keywords:** Maternal-Child Health Services (MCHS), regional health inequality, geographic health inequality, perinatal period, pregnancy, newborn

## Abstract

Objectives: The main goal of this study is to publish findings on the lifestyle factors of pregnant women in Hungary and their impact on early childhood health status by examining changes over time and regional/geographical disparities. Methods: The source of the data is the raw indicators reported by health visitors as per mandatory annual report data for the period of 1997–2019. To examine the association, we used indicators of pregnant women’s states as explanatory variables (for example, pregnant women in very late care, prenatal smoking habits, and pregnant women without care), and the outcome indicators were prematurity, intrauterine malnutrition, and newborn babies with developmental disorders. A univariate Poisson regression was used to examine the correlations. Results: Our results show a decreasing trend in the proportion of pregnant women who smoke and of pregnant women who apply late for care (after 28 weeks of pregnancy), with an increasing indicator of regional differences. The research results of the prenatal and perinatal indicators show that the counties Borsod-Abaúj-Zemplén and Szabolcs-Szatmár-Bereg are the most critical areas in terms of health status in Hungary. The number of pregnancies attended very late (after 28 weeks) and the number of women who gave birth without health visitor care are associated with the number of preterm births (R^2^ = 0.7313; *p* < 0.001; R^2^ = 0.5519; *p* < 0.001) and intrauterine growth restrictions (R^2^ = 0.3306; *p* < 0.001; R^2^ = 0.2632; *p* < 0.001). Conclusion: Interventions to improve early childhood health in some counties of Hungary are urgently needed to reduce regional disparities. Such counties include Borsod-Abaúj-Zemplén, Szabolcs-Szatmár-Bereg, Heves, Somogy, Bács-Kiskun, and Nógrád. Health education for pregnant women and activities to strengthen the compliance of pregnant women are key to improving early childhood health outcomes.

## 1. Introduction

It is a fact that in the Central-Eastern European region, including in Hungary (HU), population indicators and demographic processes have developed unfavorably in recent years. The health status of the Hungarian population, as well as that of the Central and Eastern European Countries (CEECs), closely correlates with the number of people living there. The health status of the Hungarian population is worse than in previous years, and this is especially true for men and vulnerable groups [1].

Hungary’s underperformance in examining the indicators of the subjective and objective health status of adults, as well as the health parameters of early ages (pre-, peri-, and postnatal), creates an unfavorable situation. According to the available data, infant mortality and the proportion of babies born with a low birth weight (<2500 g) are less favorable in Hungary [HU2016: 8.5% vs. EU2016: 7.6%] and maternal mortality (HU2017: 12.0 vs. EU2017: 7.6/100 live births] than in the EU) [2].

The state of health is influenced by many factors, such as those inherited, biological, and lifestyle-related, as well as social and economic effects [3,4].

Early childhood health is determined by pregnancy lifestyle (nutrition, harmful passions, smoking habits) [5,6,7,8,9,10]. According to an earlier survey, only 35.4% of pregnant women consumed fruits and vegetables with sufficient regularity. Adequate fruit and vegetable consumption among pregnant women was negatively influenced by race/ethnicity and education level [11]. Pregnant women’s health not only plays a crucial role in the health of their unborn child, but also significantly impacts health indicators in later stages of life. High infant mortality exacerbates depopulation; a low birth weight affects early survival [12]. It is now a well-known fact, supported by evidence [13], that a low birth weight is also an important risk factor for chronic adult diseases [14], such as hypertension and diabetes [15,16,17]. Prematurity and low birth weight affect weight gain in childhood, increasing the risk of cardiovascular disease in children, adolescents, and young adults, as well as retinopathy prematurity and bronchopulmonary dysplasia. But premature babies in later adulthood increase not only the risk of cardiovascular but also metabolic disorders. Premature babies experience higher blood pressure in the early stages of life, weakened blood vessel growth, increased peripheral vascular resistance, and potential cardiomyocyte remodeling. Increased weight gain in the early postnatal period may influence later body composition, promote obesity, and worsen cardiovascular outcomes. These adverse metabolic changes contribute to an increased risk of adult-onset cardiovascular events, adult-onset hypertension, and diabetes. Therefore, it would be important to continue to observe and follow up with the blood pressure monitoring of premature children and those suffering from fetal growth restriction (FGR), whose body weight percentage shows a rapid change [18].

Currently, in addition to genetics, the examination of the effects of other socio-economic processes also comes to the fore among the determining factors of an individual’s health; for example, the variables include social processes, social background, and social qualities [19,20]. According to a UNICEF survey in 2013, the rate of child deprivation in Hungary among the 29 developed countries examined was 31.9% (ranking: 27th). As a result, Hungary is one of the five countries where the proportion of children in need exceeds 25%. According to the survey, the five dimensions of children’s well-being (material well-being, health and safety, education, behavioral and risk factors, housing, and environment) are ranked 20th on average (score 18.4), which is also a critical result [21].

In Hungary, there is a well-functioning, organized, and supervised system to ensure early childhood development and a healthy childhood (Maternal-Child Health Services), which is based on health visitors working in primary health care.

## 2. Materials and Methods

The research database was based on the tables in the annual reports of health visitors between 1997 and 2019.

The data table provides numerical data for 134 types of content, all broken down by region and county. For the examined 23 years (1997–2019), it is an Excel document consisting of 253 data tables and providing data on 56776 data points. In 2019, 131 indicators were reported by health visitors working in the field. The data of the regional health visitors’ annual reports can be divided into four major groups: pregnant women; children (newborn, infant, toddler, child); the tasks performed by health visitor; and health visitor workers.

The Central Statistical Office (CSO) was responsible for the collection and publication of regional health visitor data between 1997 and 2005, which were published in the form of printed health statistics yearbooks. Between 2006 and 2019, the National Centre for Professional Surveillance and Methodology collected the data. It is now published on the website of its successor (National Directorate General of Health). Part of the data was collected by the authors from the publicly published statistical yearbooks, while the other part was obtained through formal requests to the institution responsible for data collection and management.

The COVID-19 epidemic affected the work of the health care visitor network, preventing the researchers from examining the data tables for 2020–2021, despite their availability at the time of analysis. This is because the majority of health care visitors’ personal visits and consultations occurred online, potentially distorting the data content for this period.

Microsoft Office Excel 2017 and Data Analysis and Statistical Software 13.0 (STATA 13.0) were used to analyze the indicators. Selecting the explanatory and output variables used to analyze the correlation study, a univariate Poisson regression calculation was applied using the STATA13.0 software package to examine the interaction of the different indicators. It also analyzed the direction in which the unit of the effect explanation increased, whether it induced a decrease or an increase in the output variable. The input (explanatory) variables were the pregnant woman receiving care very late, the increased need for care in pregnant women, pregnant women who smoke, and women who give birth without maternity care. The outcome indicators were premature babies, intrauterine growth restriction (IUGR), and developmental disorders. The results were considered significant if the *p*-value of a given statistical procedure was less than 0.05. In the tables presenting the results, in addition to the output and explanatory variables, the regression coefficients (b), the confidence intervals (CIs), the test strength index (R^2^), the *p*-value (*p*), and the level of significance are presented.

**Figure 1:**
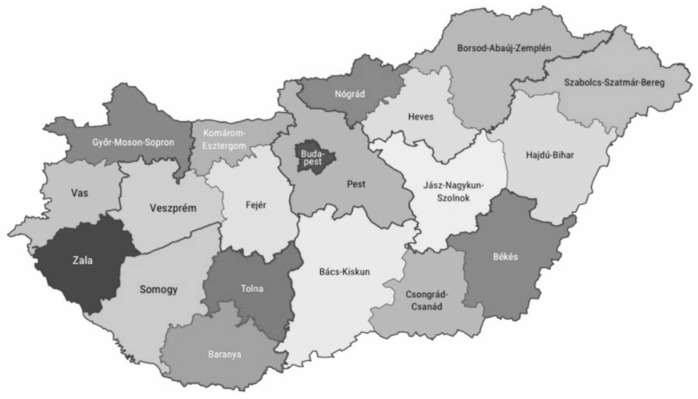
Regional Atlas—The counties of Hungary, 4 June 2020. Reprinted/adapted with permission from Hungarian Central Statistical Office. 2024, HCSO. https://www.ksh.hu/docs/teruletiatlasz/counties.pdf (accessed on 26 January 2023).

The territorial delimitation of the data analysis was conducted by the county administrative unit; it was prepared for the 19 counties of Hungary and the capital, Budapest (Figure 1).

## 3. Results

### 3.1. Prenatal Age–Pregnant Women Health Indicators, Longitudinal and Regional Differences

#### 3.1.1. Results of Long-Term Variations in Prenatal Health Status

The proportion of pregnant women in special care shows a decreasing (but not significant) trend. While, in 1997, more than 40% of pregnant women were in special care, in 2019, it fell to 37%. The proportion of pregnant women in need of special attention due to health reasons increased significantly (statistically provable) during the period under review by more than 20% (1997: 59%; 2019: 82%). In contrast to the indicator described above, the proportion of pregnant women with increased care for social reasons decreased significantly, by more than 50% (1997: 26%, 2019: 9%). The third group of causal factors for increased care includes health and social causes; at the same time, this indicator showed a significant decreasing trend as the years analyzed progressed (1997: 13%, 2019: 8%). Fortunately, the proportion of pregnant smokers also shows a declining trend over the years studied. In 1997, the rate was 17.50%, but by 2019, it had dropped to only 13%, with the national average for the 23 years examined being 14.28% (Table 1).

A slight decrease can also be observed in the case of late care. In 2019, 1% of pregnant women applied for health visitor care very late (in the third trimester). Fortunately, the still-low indicator shows a slight but decreasing trend, with the national average for the 23 years examined being 1.14 percent (Table 1).

In 1997, an expectant mother visited the health visitor’s office almost three times more frequently than in 2019. As the number of consultations increases, the number of visits decreases (Table 1).

#### 3.1.2. Results of Regional/Geographical Differences in Prenatal Health Status

The research results of prenatal indicators revealed that the county of Borsod-Abaúj-Zemplén is the most critical area for prenatal health in Hungary today, taking into account the last 23 years. The county of Borsod-Abaúj-Zemplén has the highest proportion of pregnant women requiring increased care compared to the national average, and the proportion of pregnant women cared for due to health and social reasons is also the highest. It ranks first in the county rankings in terms of the proportion of pregnant women who smoke (Table 1).

The second in the ranking is the county of Szabolcs-Szatmár-Bereg, where, in the case of the 14 examined indicators, the index of the county is among the most unfavorable in seven cases (Table 1).

Four counties also ranked third in the ranking of prenatal health indicators: the counties of Heves, Somogy, Bács-Kiskun, and Nógrád are affected. In the ranking of the analyzed 14 indicators, only the county of Bács-Kiskun managed to rank 1st among the 14 analyzed indicators, which include the proportion of women who do not receive care but are parents and the proportion of pregnant women participating in district nurse counseling (Table 1).

### 3.2. Perinatal Age, Neonates, and Health Indicators, Longitudinal and Regional Differences

#### 3.2.1. Results of Long-Term Variations in Perinatal Health Status

There is no significant change in the time course of newborns diagnosed with preterm birth, despite the fact that the proportion of newborns has decreased significantly over the past 10 years. However, the rate of preterm birth did not change (1997: 7.37%; 2019: 7.86%) according to raw indicators reported by health visitors (national average for the 23 years examined: 7.78%) (Table 2).

The proportion of newborns suffering from intrauterine growth restriction (intrauterine malnutrition, IUGR) did not change, and even increased slightly in some of the 23 years studied, before decreasing again from 2015 onwards. According to 2019 data, the national average for the 23 years examined was 2.26 percent (Table 2). There is also no change in the number of babies born with a developmental disorder. In 2019, it was 2.29%, with a slight fluctuation typical of a “sawtooth” (national average for the 23 years examined: 2.12%) (Table 2).

After returning home, the significant caring task for a health visitor is to visit the newborn’s home regularly, an indicator that has increased in recent years. The frequency of visits to the newborn’s home reached its peak in the last four years under analysis, surpassing the national average of six visits per newborn over a 23-year period (Table 2).

#### 3.2.2. Results of Regional/Geographical Differences in Perinatal Health Status

The health status around birth can be traced on the one hand to the presence or negative tendency of the health status of the previously presented pregnant mother, and on the other hand to an important indicator of the intrauterine life cycle. Upon examining the neonatal health parameters at perinatal ages, we can conclude that there is no discernible unfavorable trend. It is highlighted that the county of Somogy and Heves are also in an unfavorable position in the list of two indicators. In Somogy, the rates of premature infant births was 9.75%, with of those born with developmental disorders being 2.93%. The other county was Heves, where the number of infants born prematurely was 9.02, and the rate of intrauterine growth restriction was 3.31% (Table 2).

Of the infants born during the year, the county of Szabolcs-Szatmár-Bereg ranked second in the category of premature infants (9.11%) and had the lowest number of health visitor visits (5.65) (Table 2).

Other counties from the mentioned ranking include the following: Borsod-Abaúj-Zemplén (first place in terms of the number of intrauterine growth restriction), Nógrád (third place), and Vas, with the highest number of infants with developmental disorders, followed by Bács-Kiskun in second place. The counties of Zala and Tolna were scrutinized due to the unfavorable number of newborn visits (Table 2).

### 3.3. Analysis in Light of Maternal and Infant Health Indicators and Health Visitor Positions

#### Analysis Results Between the Conditions of Pregnant Women

During the analysis of the variables, we investigated whether a unit increase in an explanatory variable causes a change (increase or decrease) in the estimated value of the output (result) variable, while the value of all other explanatory variables remains unchanged. A Poisson regression was used to carry out this study. We used the explanatory and the output variables (preterm infants, intrauterine malnutrition, and infants born with a developmental disorder).

A measure of how many pregnant women are treated very late (after the 28th week of pregnancy) showed a positive effect on early births (R^2^ = 0.7313; *p* < 0.001), the number of babies born with intrauterine growth restriction (R^2^ = 0.3306; *p* < 0.001), and the number of children born with developmental disorders (R^2^ = 0.492; *p* < 0.001).

In the interpretation of the model (Poisson regression), this means that if the number of late-term pregnant women increases by 10.000, the number of premature births increases by 25.4, the number of intrauterine growth restrictions increases by 55.4, and the number of babies born with developmental disorders increases by 104.

The number of babies born without health visitor care, as an explanatory variable, produced similar results when examining the outcome variables. We increased the explanatory variable by 10.000 results in a positive increase in prematurity (R^2^ = 0.5519; *p* < 0.001; 22.6) and intrauterine malnutrition (R^2^ = 0.2632; *p* < 0.001; 62.8), as well as the number of births with developmental disorders (R^2^ = 0.4091; *p* < 0.001; 109.4 people).

The proportion of new mothers who smoke and receive increased care during pregnancy affects those born with developmental disorders (74.9 people and 70.5 people).

The number of unfilled health visitor positions has a positive effect (*p* < 0.001) on the number of preterm births, intrauterine growth restriction (*p* < 0.001), and disabled babies, indicating the importance of preconception care (Table 3).

## 4. Discussion

The time series analysis of the indicators reflecting pregnancy health status reveals a rapid decrease in the number of registered pregnant women from year to year. On the one hand, Hungary’s moderately high fertility rate and the fact that women of childbearing age are delaying having their first child until their thirties or beyond can explain this result. In addition to the extended maternal age, which influences the proportion of babies born with a low birth weight and determines the direction of future pregnancy care, it is significant and factual to note that the proportion of women under the age of 20 who gave birth was 6.61% [2]. Hungary has one of the highest rates in the EU for young women under the age of 20 giving birth, and 70% of teenage pregnancies are unplanned. Although parenthood may bring positive experiences for one or two young people, it is more likely to bring many negative ones with consequences. That is why it is difficult to quantify the impact on teenage years of pregnancy. After all, they are certainly influenced by one’s socio-economic situation and smoking status. There is a strong association between deprivation and conception rates in young people. After all, according to previous studies, the indicator between conception and birth rate in the poorest areas can be described as being up to six times higher than in the wealthiest areas [22]. This places an additional burden on specialist care in addition to primary care and urges the development and launch of targeted interventions.

Between 1997 and 2019, the proportion of pregnant women in special care decreased slightly, while those needing attention for health reasons significantly increased. Social reasons for increased care halved, and the rate of pregnant smokers also declined in Hungary. Regionally, Borsod-Abaúj-Zemplén is the most critical area for prenatal health, with the counties of Szabolcs-Szatmár-Bereg, Heves, Somogy, Bács-Kiskun, and Nógrád also showing unfavorable indicators. Late prenatal care and a lack of health visitor care raise the risk of preterm births, intrauterine growth restriction, and developmental disorder.

There is a slight but fortunately improving trend in the proportion of pregnant smokers and pregnant women who come to receive health visitor care very late, only after the 28th week of pregnancy, but with this national improvement, there is growing county territorial inequality (Borsod-Abaúj-Zemplén and Szabolcs-Szatmár-Bereg), typically in counties where indicators reflecting socio-economic status are also the lowest, and where the Roma population ratio is also higher [23]. The extent of the differences varied in a wide range compared to the national base number of the indicator, depending on the examined indicators, from insignificant differences to very large lags. The joint study of socio-economic factors and smoking is of paramount importance, as it is a known fact that low socio-economic status and education result in behavior that is harmful to health, such as smoking. Studies investigating the characteristics of pregnant smokers have revealed a clear impact and extent of demographic and socioeconomic determinants [8,24]. Fónai and Pénzes (2006) examined the health status of the Roma population in north-eastern Hungary and found that women (2/3) are the majority of smokers in the study population compared to men (1/3). Alcohol consumption has been observed to have the opposite effect [25].

The investigated indicators show a correlation between the number of pregnant women receiving care for very late and preterm infants, infants with intrauterine growth restriction, and those with birth defects.

A holistic treatment of premature adults’ lifelong problems (cardiometabolic) would promote their cardiovascular health, well-being, and quality of life, which could also contribute to the improvement of the nation’s public health indicators. In the current health care environment, it is challenging to recognize that preterm infants are a unique group of the population. Addressing the problems associated with preterm adults may improve the quality of life and longevity of preterm infants through clinical and research advances, the development of screening tools for early diagnosis and treatment, and long-term follow-up data for these infants [26].

Special attention must be paid to disadvantaged areas and to improving care, both in terms of funding and human resources. Depending on the indicators, it would be crucial to educate expectant mothers and babies’ parents as soon as possible [27].

It would also be necessary to establish a feedback system for health visitors who are mandated to report on their care, in order to instill in them the importance of reporting this. The incomplete data processing prevents continuous and effective feedback from being provided to the report makers [28].

After processing, the raw data systematically reported since 1997 become valuable indicators for both professional and practical implementers, but also for political decision-makers, as they map the health of pregnant women and infants in Hungary and the spatial and temporal differences found therein, which can be indispensable before planning a national strategy. Nothing supports this statement more than the results presented in this research chapter.

According to known data, the European Union and domestic social policy have launched several programs in recent years to catch up. Strategic drafts used current statistics but were not always able to synchronize with data set indicators reflecting long-term health status. Due to the complexity of the causes, regional differences call for differentiated strategies. This is especially true for the parameters characterizing the health status of pregnant women and children, as indicators characterizing the early-life stage have been shown to provide a picture of the health status of the population. Live births, infant mortality, preterm birth, low birth weight preterm infants, and congenital developmental disorders are the most important for early childhood health, and health visitors are involved in the positive development of indicators.

## Figures and Tables

**Table 1:** Summary table of descriptive analysis of prenatal health.

Indicator Groups		Analyzed Indicators National Average (%; Occasion)	Ranking of County Areas with the Highest/Worst Indicator (%; Occasion)
Prenatal period	indicators of the health status of pregnant women	pregnant women registered during the year	Budapest (capital) (17.73%) Pest c. (12.62%) Borsod-Abaúj-Zemplén c. (8.01%) Szabolcs-Szatmár-Bereg c. (6.30%)
		woman who gave birth to child	Budapest (capital) (17.66%) Pest c. (13.93%) Borsod-Abaúj-Zemplén c. (7.57%) Szabolcs-Szatmár-Bereg c. (6.29%)
		pregnant women who need require special care (40.20%)	Borsod-Abaúj-Zemplén c. (54.38%) Szabolcs-Szatmár-Bereg c. (52.43%) Somogy c. (49.89%)
		increased care: pregnant women cared for due to a health condition (68.96%)	Budapest (capital) (81.77%) Győr-Moson-Sopron c. (81.53%) Vas c. (81.25%)
		increased care: pregnant women cared for due to an environment condition (18.96%)	Szabolcs-Szatmár-Bereg c. (29.06%) Borsod-Abaúj-Zemplén c. (26.08%) Nógrád c. (23.88%) Somogy c. (23.78%)
		increased care: pregnant women cared for due to a health and an environment condition (12.08%)	Borsod-Abaúj-Zemplén c. (17.91%) Somogy c. (16.00%) Szabolcs-Szatmár-Bereg c. (15.38%)
		smoking pregnant (14.28%)	Borsod-Abaúj-Zemplén c. (24.75%) Heves c. (21.15%) Nógrád c. (20.90%) Szabolcs-Szatmár-Bereg c. (20.62%)
	quality indicators of pregnancy care	women who did not receive health visitors care but gave birth (0.54%)	Bács-Kiskun c. (0.81%) Nógrád c. (0.65%) Borsod-Abaúj-Zemplén c. (0.63%) Heves c. (0.61%) Veszprém c. (0.61%)
		pregnant women in care of health visitor, up to 12th weeks of gestation (82.83%) reverse order	Hajdú-Bihar c. (70.65%) Borsod-Abaúj-Zemplén c. (73.37%) Bács-Kiskun c. (75.51%)
		pregnant woman who health visitor cared for late, between 13th and 28th week of gestation (16.03%)	Hajdú-Bihar c. (28.09%) Borsod-Abaúj-Zemplén c. (24.91%) Bács-Kiskun c. (23.19%)
		pregnant women who health visitor cared for very late, after the 28th week of gestation (1.14%)	Borsod-Abaúj-Zemplén c. (1.72%) Szabolcs-Szatmár-Bereg c. (1.58%) Heves c. (1.45%)
		counseling sessions of health visitors per pregnant women (3.93 occasion) reverse order	Budapest (capital) (2.54 occasion) Bács-Kiskun c. (2.87 occasion) Csongrád-Csanád c. (2.99 occasion)
		visiting occasion from the health visitor per pregnant woman (4.23 occasion) reverse order	Budapest (capital) (1.97 occasion) Győr-Moson-Sopron c. (3.13 occasion) Pest c. (3.14 occasion)
		visiting occasion from the health visitor after mother gave birth child and returning home from hospital per a woman giving birth to a child (4.95 occasion) reverse order	Pest c. (4.11 occasion) Budapest (capital) (4.20 occasion) Győr-Moson-Sopron c. (4.34 occasion)

**Table 2:** Summary table of descriptive analysis of perinatal health.

Indicator Groups		Analyzed Indicators National Average (%; Occasion)	Ranking of County Areas with the Highest/Worst Indicator (%; Occasion)
Perinatal period	indicators of newborn	premature infants (7.78%)	Somogy c. (9.75%) Szabolcs-Szatmár-Bereg c. (9.11%) Heves c. (9.02%)
		infants of intrauterine growth restriction (2.26%)	Borsod-Abaúj-Zemplén c. (4.25%) Heves c. (3.32%) Nógrád c. (3.26%)
		infants with developmental disorders (2.12%)	Vas c. (3.29%) Bács-Kiskun c. (3.17%) Somogy c. (2.93%)
	quality indicators of newborn care	visiting occasion from the health visitor per newborn (6.49 occasion) reverse order	Zala c. (5.32 occasion) Tolna c. (5.13 occasion) Szabolcs-Szatmár-Bereg c. (5.65 occasion)

**Table 3:** The analysis reveals a significant correlation between the condition of pregnant women (explanatory) and preterm infants, as well as between intrauterine malnutrition and infants born with a developmental disorder (output).

Explanatory/Output Variables	Preterm Infant (Person)			R^2^	Intrauterine Growth Restriction (Person)			R^2^	Developmental Disorder (Person)			R^2^
	b *	*p*-Value	95% CI		b *	*p*-Value	95% CI		b *	*p*-Value	95% CI	
Pregnant in very late health visitor care (>28 weeks) person	25.4	<0.001	[0.0025151–0.0025083]	0.7313	55.4	<0.001	[0.0054191–0.0056556]	0.3306	104.0	<0.001	[0.0102004–0.0105997]	0.4920
Pregnant who increased care person	17.5	<0.001	[0.0017474–0.0017595]	0.7428	48.0	<0.001	[0.0047857–0.0048224]	0.5516	70.5	<0.001	[0.0070203–0.0070811]	0.5053
Smoking pregnant person	18.3	<0.001	[0.0018196–0.0018395]	0.5404	58.2	<0.001	[0.0057963–0.0058523]	0.6018	74.9	<0.001	[0.0074387–0.0075389]	0.3803
Maternity woman whithout health visitor care person	22.6	<0.001	[0.0021907–0.0023323]	0.5519	62.8	<0.001	[0.0059965–0.0065653]	0.2632	109.4	<0.001	[0.0105278–0.0113546]	0.4091
vacant position of health visitors	15.1	<0.001	[0.0014444–0.0015836]	0.1078	41.5	<0.001	[0.0039403–0.004356]	0.0828	46.1	<0.001	[0.0042699–0.0019554]	0.0438

* per 10,000 newbor, position.

## Data Availability

The authors declare that the data are open access, they are available to anyone and can be requested. https://www.nnk.gov.hu/index.php/egeszsegugyi-igazgatasi-foosztaly/vedonoi-tevekenyseg/modszertan/1265-teruleti-vedonoi-jelentes.html (accessed on 26 January 2023).
